# Supplementary material for: Spatial epidemiology and adaptive targeted sampling to manage the Chagas disease vector Triatoma dimidiata
Source: PLoS Negl Trop Dis. 2022 Jun 2;16(6):e0010436. doi: 10.1371/journal.pntd.0010436 (PMC9162375; doi:10.1371/journal.pntd.0010436)
Supplement: S1 Table — (PDF) [file pntd.0010436.s001.pdf]

Table S1 Additional information from the 2011 EcoHealth survey.

Village	Total number houses	Number houses after preparation	Infestation rate (after preparation)
El Amatillo	215	172	15.3%
El Cerrón	205	147	36.7%
El Guayabo	302	251	33.3%
El Paternito	138	108	38.3%
La Prensa	280	207	38.8%
Total	1,140	885	32.3%
