# Supplementary material for: Spatial epidemiology and adaptive targeted sampling to manage the Chagas disease vector Triatoma dimidiata
Source: PLoS Negl Trop Dis. 2022 Jun 2;16(6):e0010436. doi: 10.1371/journal.pntd.0010436 (PMC9162375; doi:10.1371/journal.pntd.0010436)
Supplement: S2 Table — (PDF) [file pntd.0010436.s002.pdf]

Table S2 Socioeconomic variables used for model fitting.

House factors	Values/range
Bed hygiene	Good, poor
Sign of bird nests inside	Yes, no
Location of chicken coop	Inside or adjacent to house, outside house, none
Clutter in bedroom	Yes, no
Poorly lit bedroom	Yes, no
Floor material	Dirt, other
Piles of construction material	Adobe or clay, wood or metal, none
Firewood location	Inside house, directly outside, outside, none
Grain storage in house	Yes, no
House age	Less than one year, 2-6 years, more than 7 years
House hygiene	Good, poor
Kitchen location	Inside house, outside, shared or none
Land for agriculture	Rented, owned, none
Sign of rats	Yes, no
Sign of other small animals	Yes, no
Bedroom wall condition	Good, deteriorated
Wall condition throughout home	Good, deteriorated
Material house wall	Adobe, bajareque, palopique, brick or other
Material roof	Aluminum or cement, clay or vegetal material, nylon panels
Windows in bedroom	Yes, no
Number residents	1-15
Number chickens	0-60
Number dogs	0-12
Number pigs	0-12
