# Supplementary material for: Spatial epidemiology and adaptive targeted sampling to manage the Chagas disease vector Triatoma dimidiata
Source: PLoS Negl Trop Dis. 2022 Jun 2;16(6):e0010436. doi: 10.1371/journal.pntd.0010436 (PMC9162375; doi:10.1371/journal.pntd.0010436)
Supplement: S1 Appendix — (PDF) [file pntd.0010436.s003.pdf]

Appendix S1 Mathematical definitions.

Matérn covariance function

For any pair of points at distance d from each other, the Matérn covariance between these points is

$$C(d) = \frac{\sigma_s}{2^{\nu-1}\Gamma(\nu)}(\kappa d)^{\nu} K_{\nu}(\kappa d),$$

where Γ is the Gamma function and K_ν the modified Bessel function. The parameters σ_s and ν are the spatial standard deviation and smoothness, respectively, while κ is implicitly defined via the effective range $\rho = \sqrt{8\nu}/\kappa$, which is the distance at which the correlation between points roughly becomes 0.1.

Deviance information criterion and marginal likelihood

Let $\mathcal{M}$ denote a statistical model of interest. The deviance information criterion of $\mathcal{M}$ is

$$D(\bar{\boldsymbol{\theta}}) + 2p_D,$$

where $D(\theta) = -2\log(p(\boldsymbol{y} \mid \theta))$ is the deviance, $\bar{\boldsymbol{\theta}}$ the posterior expectation of $\mathcal{M}$, and p_D the effective number of parameters.

The marginal likelihood is

$$p(\boldsymbol{y} \mid \mathcal{M}) = \int p(\boldsymbol{y} \mid \mathcal{M}, \boldsymbol{\theta}) p(\boldsymbol{\theta} \mid \mathcal{M}) d\boldsymbol{\theta}.$$
