# Supplementary material for: Spatial epidemiology and adaptive targeted sampling to manage the Chagas disease vector Triatoma dimidiata
Source: PLoS Negl Trop Dis. 2022 Jun 2;16(6):e0010436. doi: 10.1371/journal.pntd.0010436 (PMC9162375; doi:10.1371/journal.pntd.0010436)
Supplement: Attachment — Submitted filename: Case_etal2022_answer.pdf [file pntd.0010436.s006.pdf]

Spatial epidemiology and adaptive targeted sampling to manage the Chagas disease vector Triatoma dimidiata

B. K. M. Case, Jean-Gabriel Young, Daniel Penados, Carlota Monroy, Laurent Hébert-Dufresne, Lori Stevens

April 14, 2022

Dear Editor and Reviewers,

We thank the referees for their thoughtful reports. The main changes to our manuscript in response to the feedback are:

- We have added additional information about the climate and land use of the study area, the original survey which produced the data we used, and our data preparation methods. This includes the addition of Table S1, which provides the number of housing structures recorded in each village prior to data cleaning.
- We have created a publicly available repository which contains the processed data, clearly commented code, and a brief tutorial on spatial modeling (found at `https://github.com/brendandaisy/chagas-adaptive-sampling/tree/plos-ntd`).

Please find below a point-by-point response below. We also include a `diff` version of the manuscript to highlight the modifications.

The authors.

Reviewer 1

Methods

Comment 1.1

The presented methods are well described and support the proposed objectives. The methods are well organized and easy to understand. The information was presented in a simple and transparent way.
Yes. The objectives of the study are clearly articulated with the hypothesis. Yes. The study design is appropriate. No. The population is not clearly described. More details in the "Summary and General Comments" section. Yes. The sample size is sufficient to ensure adequate power to address the hypothesis being tested. Yes. The statistical analysis used to support conclusions was clear.

Reply 1.1

Thank you, we are glad the methods were generally easy to follow. Please see below for our responses regarding the study population.

Results

Comment 1.2

The results are well presented. The ideas and information presented are sequential and logical. The results are well supported by informative figures and tables.
Consider better describe the topic "simulation results" and explain the reason why the proposed model "produce designs, which failed to satisfy the design target of reducing the true infestation rate below 5
The analysis presented matches the analysis plan. The results are clearly and well presented. The figures are informative, and in a sufficient quality.

Action taken 1.2

To add clarity to the simulation results section, we changed the titles of the simulation study methods and results sections to be more informative. We also added the sentence "This highlights the importance of exploring the design space during early stages of sampling, in order to mitigate prediction bias" on line 317, for some brief reasoning behind why less exploratory versions of the proposed strategy can fail to satisfy the design target, before this is expanded upon in paragraphs 3 and 4 of the Discussion.

Conclusions

Comment 1.3

The article suggests new options for implementing long-term T. dimidiata vector control. Describe an adaptive strategy for public health interventions, which transitions from prioritizing areas of greatest uncertainty to those perceived to be most at risk, using data from five villages in southeastern Guatemala.
This is an interesting approach bringing relevant strategies aiming to interrupt disease transfer, targeting only a subset of house community. Also, demonstrate how sampling strategies could compliment the EcoHealth initiative to conserve insecticide, the time of health personnel and other important approaches.

The conclusions are supported by the data presented. The limitations of analysis are clearly described. The authors discuss well how these data can be helpful.

Reply 1.3

We are glad the approach was found relevant and of possible use to the EcoHealth initiative.

General Comments

Comment 1.4

The main objective of this research was duly achieved, the methodologies and results are duly described and discussed. Congratulations. Despite that, as a neglected population disease, it is important to bring relevant techniques, such as those presented by the authors in this publication, to governments and vulnerable communities accessibly. As a suggestion, evaluate the possibility of including in the discussion of this manuscript the necessity of developing a tool (system or application), widely accessible, to help regional leaders and government to implement spatial geostatistical models, helping mitigate the reality of these communities, in a more accessible way. Alternatively, suggesting the validation of the proposed methods in other countries will help achieve breadth and certification of the methods widely.

Action taken 1.4

We thank the reviewer for their kind words. We have since taken a number of steps to ensure our methods are reproducible and accessible. All the data and code needed to replicate our analysis is now available in a public GitHub repository which has been linked to in the paper. This includes the processed data we used as input for our results, with anonymized geocoordinates, as well as a brief tutorial on spatial modeling and some examples of using our code. Additionally, at the end of the second-to-last paragraph in the Discussion, we have added the sentence ”Finally, additional research should go into the development of tools for adaptive, targeted intervention strategies, and how such software can best be applied for the direct benefit of the community.”

Comment 1.5

The manuscript need to better describe the study population and the sampling methods;
The manuscript should inform the proportion of ”missing data” removed of the study, for better understanding of readers, taking into consideration the analysis of this proportion by each location.

Action taken 1.5

Additional information about the study region, sampling methods, proportion of missing data, and reasons for removing observations have been added to the description of our methods (Section 2.1 ”Data preparation”). We have also added Table S1 to summarize the number of observations before/after data cleaning, as well as the infestation rate in each village. For convenience, we include this table below:

Village	Total number houses	Number houses after preparation	Infestation rate (after preparation)
El Amatillo	215	172	15.3%
El Cerrón	205	147	36.7%
El Guayabo	302	251	33.3%
El Paternito	138	108	38.3%
La Prensa	280	207	38.8%
Total	1,140	885	32.3%

Comment 1.6

Line 8 – Suggestion of change: Triatomine levels – triatomine infestation levels.

Reply 1.6

This was changed.

Comment 1.7

Line 11 – Better develop the idea of seroprevalence in this sentence. Is this the seroprevalence of domestic animals, of humans? Please, be more informative.

Reply 1.7

Changed to "...sufficient to eliminate *T. cruzi* seroprevalence in children aged 6 months to 15 years."

Comment 1.8

Line 16 – Suggestion of change: ... is endemic to the continent and infests peridomestic and sylvatic environments ... – ... is endemic to the continent, living at sylvatic and infesting peridomestics (domestic?) environments...

Reply 1.8

Changed to "...endemic to Central America, living in peridomestic and sylvatic as well as domestic environments."

Comment 1.9

Line 114 – The manuscript does not explain numbers of "missing information" and how much it represents the total and by each location

Reply 1.9

The number and proportion of data points before preparation is now available in Section 2.1 and Table S1.

Comment 1.10

Line 118 – Do the team member follow some specific protocol to search the houses and find the T. cruzi vectors?

Reply 1.10

More information about the protocol used to search houses can now be found in the final paragraph of Section 2.1.

Reviewer 2

Comment 2.1

The manuscript is well written, with objectives consistent and consolidated hypothesis.
The results are clear and the figures and tables are of sufficient quality for clarity.
The manuscript "Spatial epidemiology and adaptive targeted sampling to manage the Chagas disease vector Triatoma dimidiata" brings some important challenges for entomological surveillance, as the operational capacity of the service, to reduce the proportion of infected households with Triatoma dimidiata in short time.

Reply 2.1

We are glad you found the manuscript satisfactory. Thank you for taking the time to review our manuscript.

Reviewer 3

Comment 3.1

The objectives of the study were clearly articulated with a clear and testable hypothesis. The study design is appropriate to address the exposed goals. The studied population was sufficiently described in the manuscript and the size in the sample was sufficient for the actual tests. Good geostatistic solutions were found for the resolution of study questions.
The results were very well presented and in total sync with the objectives of the study.
The conclusions of the study were correctly placed. Do not extrapolate the capacity of the study. Leaving the goal of the study to be another useful tool in combating Chagas in small communities. The authors were also quite clear in the study limitations and the need for new developments and approaches.

Reply 3.1

Thank you for such kind words! We appreciate you spending the time to review our manuscript.

General Comments

Comment 3.2

First I would like to congratulate the work presented. Demonstrated methodological clarity and alignment with the very significant goals. It was also written very clearly. That said I would like to put some suggestions that I believe can enrich work or future unfolding.

Reply 3.2

Thank you for the constructive feedback. Please find responses to specific items below.

Comment 3.3

The authors selected socioeconomic variables all linked to the domicile and peridomicile. Surely the use of indicators expressing the ecological condition of the surroundings of households and the village will enrich the work.

Action taken 3.3

While this information was not available in the dataset we used, it would certainly be interesting to investigate whether such environmental variables could serve as an alternative source of information. We have therefore added the sentence ”It would also be interesting to investigate the use of additional environmental variables as an alternative to the socioeconomic information, which may provide similar gains in sampling efficiency while being easier to collect” to the second-to-last paragraph in the Discussion.

Comment 3.4

The vectors despite being well adapted to the human domicile can have in the external areas of the domicile and the ecological niches that operate from reservoirs for future infestations. In addition to the ecological indicators I believe that the description and the preparation of cultural indicators of the locations and residents of households would be very relevant. They are certainly difficult to operational.

Reply 3.4

Although several cultural indicators were collected as part of the original survey, we ultimately did not use most of them in our analyses since they were either directly related to the infestation status of the home, or there was insufficient variety in the responses to be used for statistical analysis.

Comment 3.5

In both cases if it is not possible to control the future study of these types of ecological and cultural indicators that would approach the study of the theoretical reference presented in it. Then at least make a description of the ecological framework and culture from the area such as percentage of vegetated area, rainfall etc.

Action taken 3.5

We have added some additional information regarding the environment of the study area in Section 2.1, including the climate and land use in the surrounding area.

Comment 3.6

Finally I suggest that they approach the environmental problems intrinsic to the use of insecticide to the environment as well as the selection of resistant individuals in the population.

Action taken 3.6

We now mention insecticide resistance as another challenge of *T. dimidiata* control in the second paragraph of the Introduction.
