# Supplementary material for: Spatial epidemiology and adaptive targeted sampling to manage the Chagas disease vector Triatoma dimidiata
Source: PLoS Negl Trop Dis. 2022 Jun 2;16(6):e0010436. doi: 10.1371/journal.pntd.0010436 (PMC9162375; doi:10.1371/journal.pntd.0010436)
Supplement: Attachment — Submitted filename: Manuscript number.docx [file pntd.0010436.s005.docx]

Manuscript number: PNTD-D-21-01764

The manuscript "Spatial epidemiology and adaptive targeted sampling to manage the Chagas disease vector *Triatoma dimidiata*" brings some important challenges for entomological surveillance, as the operational capacity of the service, to reduce the proportion of infected households with *Triatoma dimidiata* in short time.

The manuscript is well written, with objectives consistent and consolidated hypothesis.

1)	What are the traditional methods and the additional measures adopted for the vector control?

2)	How many professionals were necessary to collect the information in 885 houses? How long were them take time for it? (Line 116)

3)	Of the 885 houses, how many were positive for Chagas’s vector? Is the value being high or low to compare with the models' results?
